# Post-intubation Tracheoesophageal Fistula Following Meningioma Excision: A Case Report and Literature Review

**DOI:** 10.7759/cureus.83748

**Published:** 2025-05-08

**Authors:** Nayef Alkhalil, Abed AlRaouf Kawtharani, Charles Nahas, Antoine Abou Rached

**Affiliations:** 1 Gastroenterology Division, Lebanese University - Faculty of Medical Sciences, Beirut, LBN; 2 Pulmonology Division, Hôpital du Sacré-Cœur, Beirut, LBN; 3 Internal Medicine Department, Gastroenterology Division, Lebanese University - Faculty of Medical Sciences, Beirut, LBN

**Keywords:** coughing, iatrogenic complication, post-intubation complications, recurrent aspiration pneumonia, tracheo-esophageal fistula

## Abstract

A tracheoesophageal fistula (TEF) is defined as a pathological connection between the trachea and the esophagus, leading to a spillover of oral and gastric secretions into the respiratory tract. TEF is either congenital or acquired. Congenital TEFs are rare in adults and are mainly associated with esophageal atresia that presents during infancy and childhood. However, much less is known about acquired TEF, which mainly affects the adult population. In adult patients, the majority of TEFs are acquired, with mediastinal malignancies, such as esophageal cancer and lung cancer, representing the etiology in over half the cases. The principal non-malignant causes of acquired TEFs include post-intubation trauma, chronic infections (e.g., tuberculosis), radiation injury, and post-surgical complications. Because they are rarely encountered in adults, TEFs are often difficult to diagnose and can present with nonspecific signs and symptoms. Endoscopic and surgical measures can be undertaken to prevent life-threatening complications and malnutrition. In this article, we report the case of a 68-year-old female patient who developed a large TEF after prolonged intubation in the ICU post undergoing a craniotomy for meningioma excision.

## Introduction

A tracheoesophageal fistula (TEF) is an abnormal connection between the trachea and esophagus, resulting in the spillage of oral and gastric secretions into the airway system. TEFs are categorized as either congenital or acquired. Congenital TEFs are rare in adults, typically associated with esophageal atresia presenting in infancy and childhood, whereas acquired TEFs are more common in adults [1]. In adults, acquired TEFs often arise from mediastinal malignancy (e.g., esophageal cancer, lung cancer), accounting for over half of cases [2]. Acquired non-malignant TEFs can result from chronic infectious processes (e.g., tuberculosis), radiation damage, post-intubation trauma and post-surgical complications [3]. Due to their infrequent occurrence in adults, TEFs are often challenging to diagnose and may present with nonspecific signs and symptoms. Endoscopic and surgical interventions are utilized to prevent life-threatening complications and malnutrition [4]. In this article, we report the case of a 68-year-old female patient who underwent a craniotomy for meningioma excision and developed a large TEF after prolonged intubation in the intensive care unit (ICU).

## Case presentation

A 68-year-old female patient with a known history of hypertension, dyslipidemia, and a recent craniotomy for meningioma excision (four weeks prior) was admitted to the internal medicine ward for severe aspiration pneumonia. The gastroenterology team was consulted due to recurrent aspiration pneumonia following her craniotomy.

On history-taking, the patient reported having dysphagia and persistent regurgitation of solids and liquids that started after her craniotomy and extubation. Physical examination revealed a conscious, cooperative, and oriented patient with a soft, non-tender abdomen and no palpable neck masses. However, she experienced immediate coughing and shortness of breath upon attempting a trial of swallowing small sips of water. Laboratory results on admission are summarized in Table 1.

**Table 1:** Laboratory test results of the patient in the emergency department (ED)

Lab Test	Result	Reference Range
White blood cells (WBCs) count	14,820	4,500-11,000/mm³
Hemoglobin	11.9	12.5-16.5 g/dL
Platelet count	115000	150,000-450,000/mm³
Creatinine	0.8	0.6-1.2 mg/dL
Blood urea nitrogen (BUN)	27	7-20 mg/dL
Sodium	142	135-145 mEq/L
Potassium	3.9	3.5-5 mEq/L
Chloride	106	95-105 mEq/L
Bicarbonate	33	22-28 mEq/L
C-reactive protein (CRP)	98.1	0-6 mg/L
Alanine transaminase (ALT)	31	10-33 U/L

**Figure 1:**
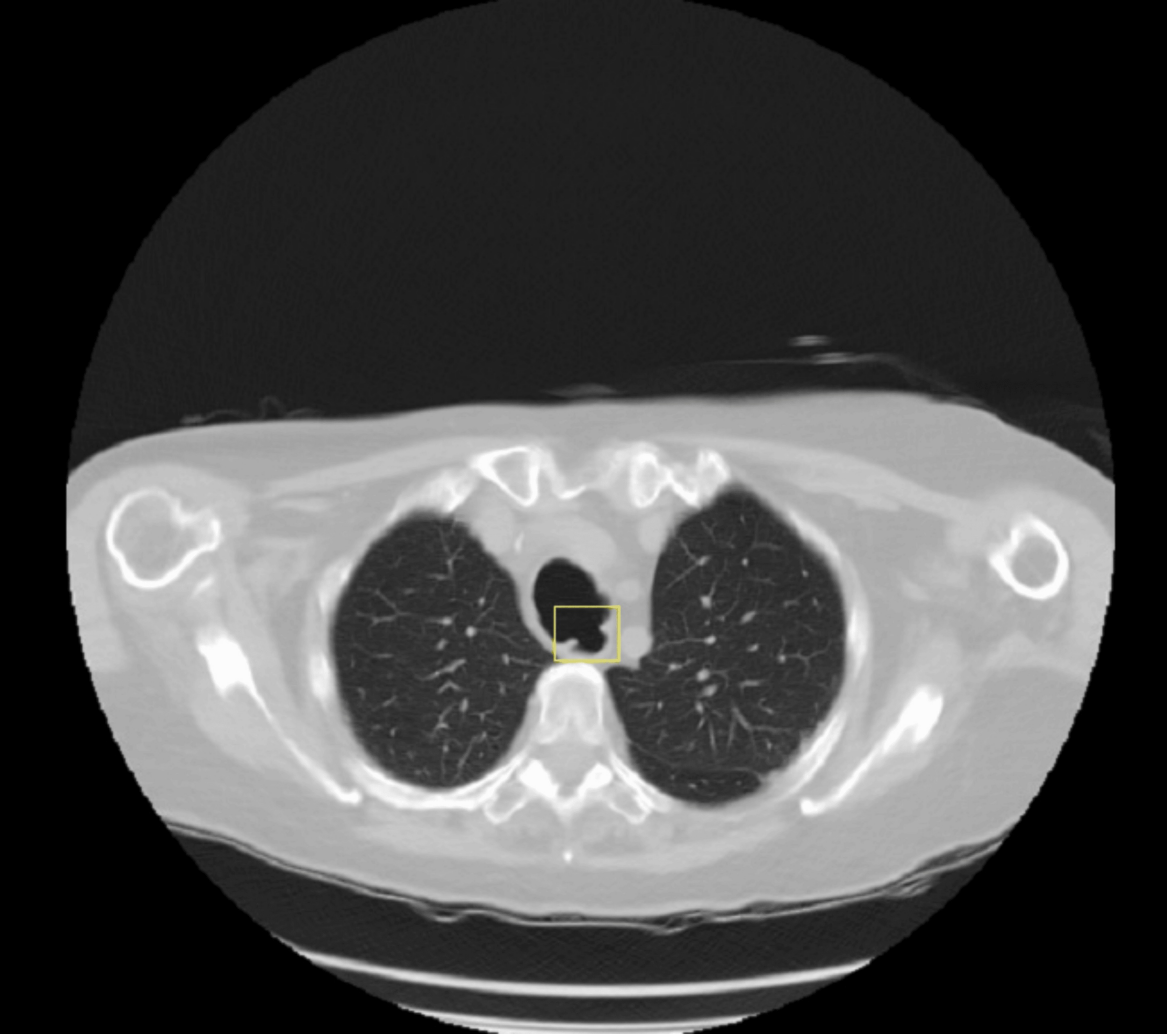
Axial view of non-enhanced CT Chest (Lung window) showing abnormal connection (yellow box) between the trachea (superior structure) and the esophagus (inferior structure), compatible with TEF TEF: tracheoesophageal fistula

Initial non-contrast chest CT revealed a wide proximal tracheoesophageal communication (Figure 1). However, a subsequent Gastrografin swallow study was inconclusive because of the proximal position of the fistula, which was obscured by bony structures overlying it (mandible, etc.), but it showed tracheal dilatation at the cervico-thoracic region. Given the patient's history of prolonged endotracheal intubation (eight days in the ICU post-craniotomy) and persistent respiratory symptoms, a TEF was strongly suspected, alongside other potential neurological causes of dysphagia. Esophagogastroduodenoscopy (EGD) was, therefore, performed.

**Figure 2:**
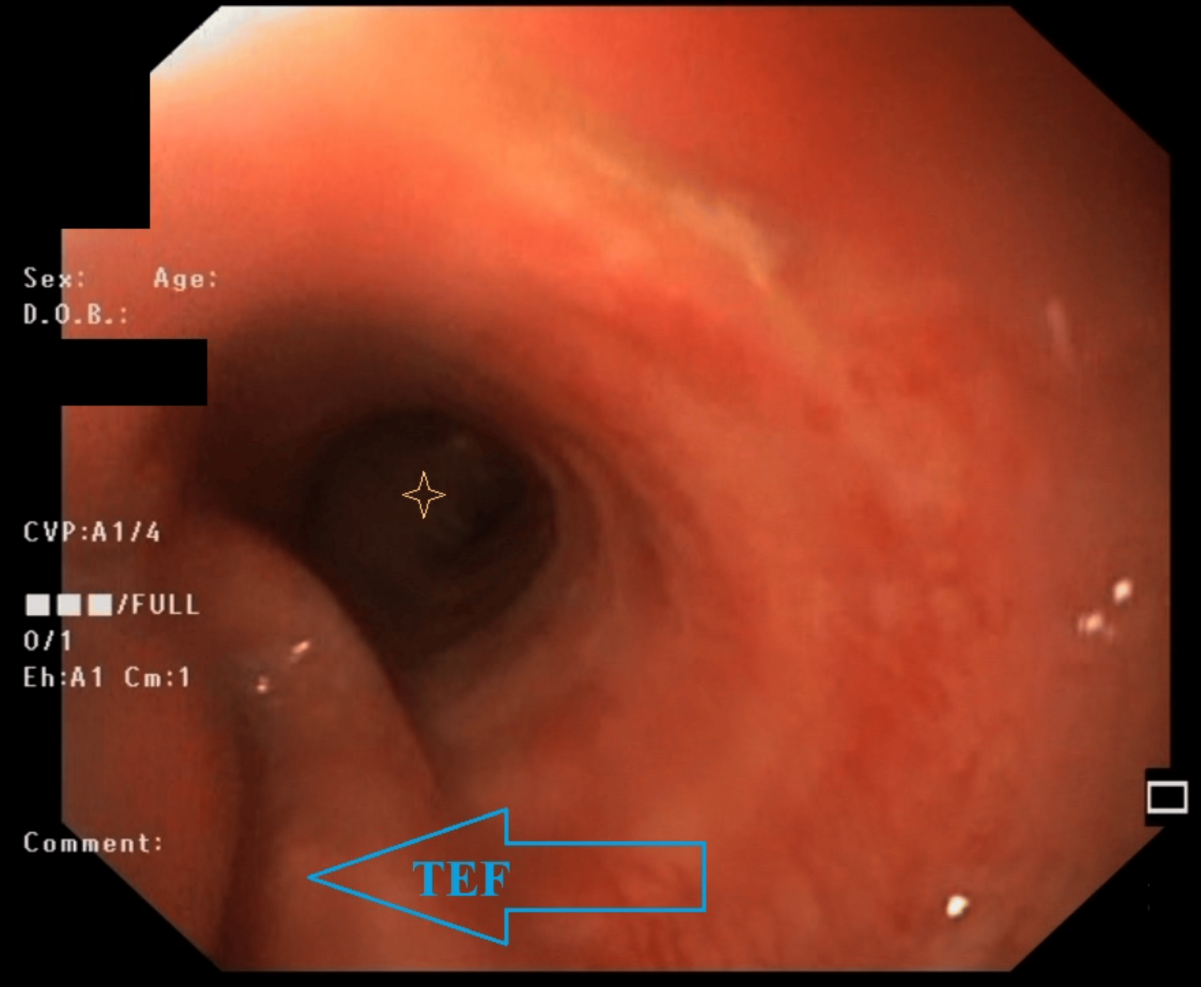
EGD view of the TEF (blue arrow) directly after bypassing the upper esophageal sphincter The carina (bronchial bifurcation) is evident distally in the view (marked by the yellow star/asterisk). TEF: tracheoesophageal fistula; EGD: esophagogastroduodenoscopy

EGD revealed a large (> 5 cm) TEF in the upper third of the esophagus directly after bypassing the upper esophageal sphincter (Figure 2). Percutaneous endoscopic gastrostomy (PEG) tube placement was performed during the same gastroscopy to initiate enteral feeding.

**Figure 3:**
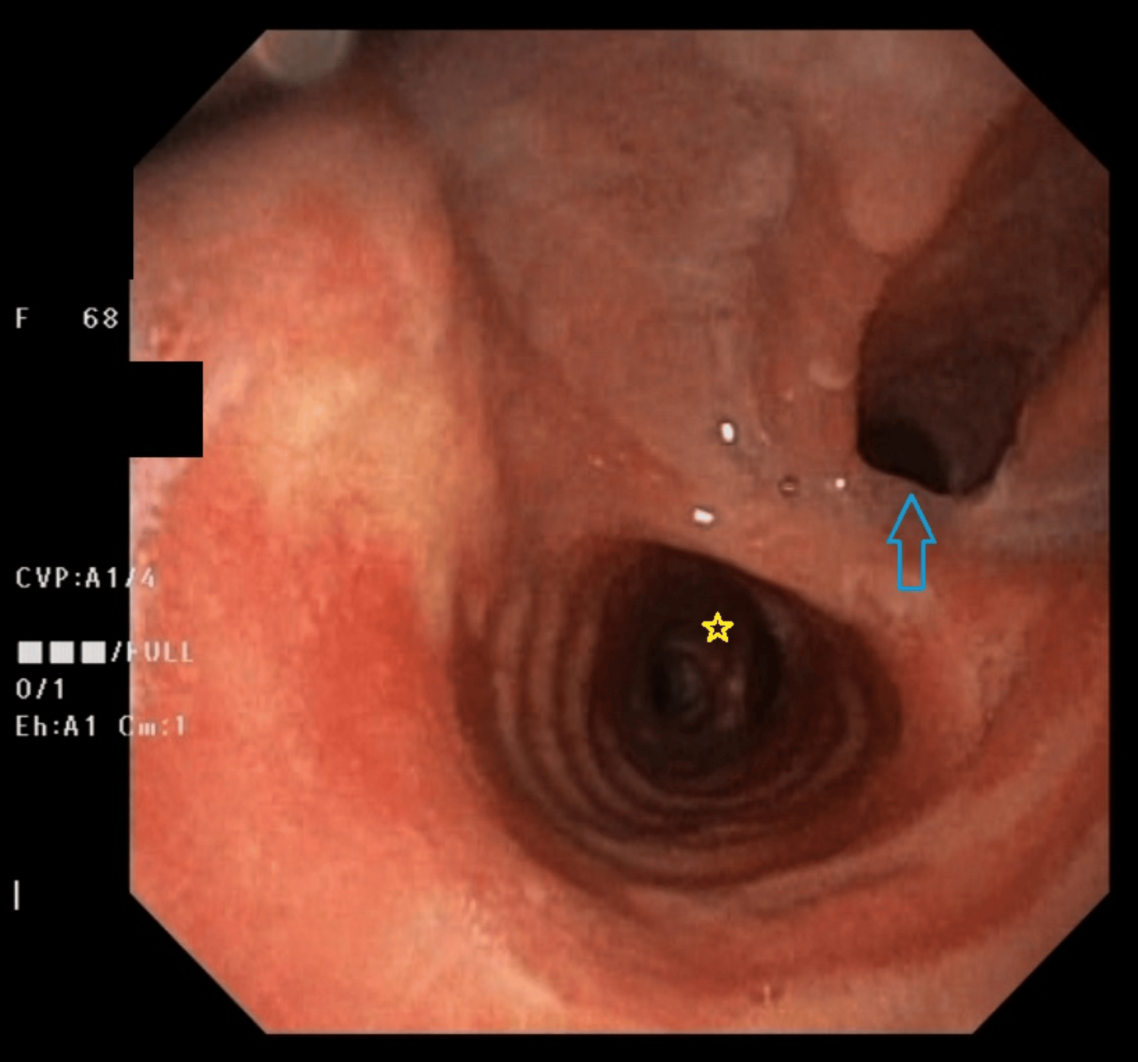
Bronchoscopic view of the TEF (pointed at by the blue arrow) The tracheal carina is evident distally in the view (marked by the yellow star). TEF: tracheoesophageal fistula

Subsequently, a bronchoscopy was performed to exclude tracheal or bronchial malignancy. This revealed no evidence of malignancy but confirmed the large TEF (Figure 3).

The patient was referred to another medical institution under the care of a specialized thoracic surgery team with advanced expertise in handling such cases.

## Discussion

Acquired TEF represents a pathological connection between the trachea and esophagus, resulting from various disease processes. These processes are categorized as malignant or benign. In adults, malignant etiologies are responsible for over 50% of acquired TEFs [3]. Benign causes include post-intubation trauma, chronic granulomatous infections (for example, tuberculosis), radiation damage, and post-surgical complications [3].

Historically, granulomatous mediastinal infections, such as tuberculosis, were the predominant cause of benign acquired TEFs. However, the increased prevalence of prolonged mechanical ventilation and tracheostomies has resulted in iatrogenic causes accounting for approximately 75% of benign TEFs [1]. Diddee and Shaw estimated that up to 3% of mechanically ventilated patients develop TEF due to cuff-related injury [2]. Given that the pathophysiology of injury is primarily due to "pressure necrosis" from the inflatable balloon, tracheostomies do not appear to reduce the occurrence of TEF when compared to endotracheal tubes. Other iatrogenic etiologies include aggressive, harmful intubation and secretions suctioning. Chronic medical conditions, such as diabetes mellitus, recurrent respiratory infections, corticosteroid use, and the presence of nasogastric tubes, further increase the risk of TEF formation associated with mechanical ventilation [2].

The clinical presentation of TEF varies according to the speed of fistula formation, size, location, patient's medical condition, and immune status. In a study of over 200 patients, BURT et al. reported the most common clinical manifestations as coughing (56%), aspiration (37%), pyrexia (25%), difficulty swallowing (19%), pneumonia (5%), hemoptysis (5%), and chest discomfort (5%) [5]. Ono’s sign (paroxysmal coughing triggered by swallowing of fluids or food) is present in 81% of patients with known TEF, albeit it exhibits low sensitivity and specificity [1]. The average time from symptom onset to diagnosis of malignant TEF is approximately 7.3 ± 4.25 months (range 1-58 months). The presentation of symptoms in benign TEFs exhibits greater variability, with traumatic etiologies typically manifesting within 5 to 15 days, whereas iatrogenic cuff-related injuries may present between 21 and 30 days [1,2,4].

The diagnosis of tracheoesophageal fistula relies on a combination of thoracic imaging and endoscopic evaluation, including upper endoscopy (EGD) and, when feasible, flexible bronchoscopy. Initial assessment of respiratory symptoms with chest radiography is a pragmatic approach; however, this modality often lacks definitive TEF-specific findings and may instead reveal secondary changes such as basal infiltrates or mediastinal widening. A chest computed tomography (CT) scan can further delineate fistula characteristics, aerodigestive tract anatomy, and mediastinal pathology. Nevertheless, the sensitivity and specificity of CT in confirmed TEF cases remain unestablished [1].

While no formal guidelines handling this topic exist, expert consensus emphasizes the necessity of esophagography and endoscopy for both diagnostic confirmation and preoperative assessment in suspected TEF cases. Contrast-enhanced esophagography is reported to detect the fistula in approximately 70% of affected patients [1].

Although esophagography is suboptimal for sedated or mechanically ventilated patients, endoscopy (via either gastroscopy or bronchoscopy) remains the preferred diagnostic modality for both awake and anesthetized individuals. Endoscopic visualization facilitates precise localization, sizing, and characterization of the TEF and can be conducted under moderate sedation or general anesthesia [1].

Spontaneous closure of TEFs is rare, and without treatment, the outcome is often poor, with survival measured in weeks. Management necessitates a multidisciplinary approach involving thoracic surgery, oncology, gastroenterology, and interventional pulmonology specialists [6]. Due to limited data, there is a lack of consensus and established guidelines for TEF management, resulting in significant variation in clinical practice [6].

The cornerstone of preoperative management involves mitigating complications secondary to the anatomical defect while addressing modifiable risk factors for fistula persistence. A critical consideration is preventing respiratory tract contamination, which may precipitate pneumonitis or pulmonary sepsis. Therapeutic measures include acid-suppressive therapy to decrease gastric acid secretion and volume. Mechanical interventions, such as maintaining ≥45° head-of-bed elevation, enforcing nil per os status, and implementing frequent oropharyngeal suction complement pharmacological management. For mechanically ventilated patients, advancing the endotracheal tube to position the cuff below the fistula site may minimize the pulmonary aspiration risk. To reduce pressure-induced necrosis at the fistula site, removal of nasogastric and orogastric tubes is recommended, particularly in intubated individuals. In specific cases, gastrostomy tube placement for gastric decompression and jejunostomy tube insertion for enteral nutrition may be warranted [1].

Regarding invasive treatment options, surgery with curative intent is generally performed for benign TEF in surgically eligible patients, while palliative management is typically reserved for malignant TEFs and surgically ineligible patients [6]. The treatment of benign TEFs aims for cure, primarily through surgery, although not all cases are suitable. Surgical repair, a technically demanding procedure, may involve various approaches depending on fistula size and is associated with not only a 75-94% success rate in fistula closure but also significant complication rates (up to 50%). For patients who are not surgical candidates or have unsuitable lesions, palliative or local therapies are considered. Palliative interventions for larger fistulas (> 5 mm) often involve stenting, whereas smaller fistulas (≤ 5 mm) may be treated with local therapies, such as endoscopic clip placement or occlusive techniques, which have certain limitations [7].

This case describes a TEF that developed as a complication of prolonged intubation in an intensive care setting following meningioma resection. The patient, a female in her seventh decade of life, exhibited characteristic symptoms, including cough, dysphagia, and recurrent aspiration pneumonia requiring multiple hospitalizations within four weeks post-extubation. This clinical timeline aligns with existing literature on post-intubation TEF development [1,2,4].

Diagnostic evaluation revealed limitations of esophagography in fistula identification, while chest CT demonstrated superior diagnostic utility by clearly visualizing the tracheoesophageal communication. Confirmatory diagnosis was ultimately achieved through combined gastroscopic and bronchoscopic evaluation.

Given the extensive defect size precluding endoscopic management, the patient was transferred to a tertiary care center with specialized surgical expertise. This case highlights both the diagnostic challenges in TEF evaluation and the need for further studies comparing the sensitivity of various imaging modalities in fistula detection. Additionally, it underscores the importance of establishing standardized protocols for managing complex cases requiring multidisciplinary care and advanced surgical intervention.

## Conclusions

An acquired tracheoesophageal fistula (TEF) remains a serious complication of prolonged mechanical ventilation, with diagnostic and therapeutic challenges persisting despite advances in imaging and endoscopy. This case highlights the importance of early recognition in high-risk patients, particularly given the wide range of its causes (infectious, iatrogenic, etc.). While surgical repair offers the best chance of cure, not all patients are candidates, underscoring the need for individualized, multidisciplinary management. Future efforts should focus on standardizing diagnostic protocols, refining preventive strategies for cuff-related injuries, and developing evidence-based treatment algorithms.

This case serves as a compelling reminder of the delicate balance between life-sustaining interventions and their potential sequelae, urging continued collaboration among intensivists, surgeons, and radiologists to advance the care of patients with TEF. As critical care advances, so too must our vigilance in preventing and managing its complications, a challenge that demands both technical innovation and systemic introspection.

## References

[1] Kim HS, Khemasuwan D, Diaz-Mendoza J, Mehta AC (2020). Management of tracheo-oesophageal fistula in adults. Eur Respir Rev.

[2] Diddee R, Shaw IH (2006). Acquired tracheo-oesophageal fistula in adults. BJA Educ.

[3] Shah SJ, Jadhav UE, Agrawal DP (2022). Acquired tracheo-esophageal fistula in adult-a classical case of 'what not to do'. Indian J Thorac Cardiovasc Surg.

[4] Hasan L, Sharma B, Goldenberg SA (2022). Acquired tracheoesophageal fistulas: a case report and review of diagnostic and management challenges. Cureus.

[5] Burt M, Diehl W, Martini N, Bains MS, Ginsberg RJ, McCormack PM, Rusch VW (1991). Malignant esophagorespiratory fistula: management options and survival. Ann Thorac Surg.

[6] Bibas BJ, Cardoso PF, Minamoto H, Pêgo-Fernandes PM (2018). Surgery for intrathoracic tracheoesophageal and bronchoesophageal fistula. Ann Transl Med.

[7] Siboni S, D'Aiello AF, Chessa M, Bonavina L (2022). Tailored endoscopic treatment of tracheo-oesophageal fistula using preoperative holographic assessment and a cardiac septal occluder. BMJ Case Rep.

